# Molecular Engineering Strategies for Organic Pre‐Sodiation: Progress and Challenges

**DOI:** 10.1002/advs.202500906

**Published:** 2025-03-07

**Authors:** Zhijian Cai, Huai Chen, Fujun Niu

**Affiliations:** ^1^ School of Advanced Energy Sun Yat‐sen University (Shenzhen) Shenzhen 518107 China

**Keywords:** functional group modification, molecular engineering, orangic pre‐sodiation, potential control, structure‐activity relationship

## Abstract

Pre‐sodiation, which is capable of supplying additional active sodium sources to sodium‐ion batteries (SIBs), has been widely accepted as one of the most promising approaches to address the issue of active sodium loss during initial charging and subsequent cycling. Organic materials, with their design flexibility and abundant sources, are well‐suited for large‐scale applications. To achieve effective organic pre‐sodiation, precise control over reaction potential is essential. In view of this, molecular engineering strategies are developed to mediate the pre‐sodiation potential of organic materials for efficient pre‐sodiation. Nevertheless, a comprehensive review of molecular engineering in organic pre‐sodiation is still lacking. This timely review aims to present the crucial role of molecular engineering in organic pre‐sodiation and provide an up‐to‐date overview of this field. After the showcasing of fundamental details of molecular engineering in organic pre‐sodiation, recent advances in modifying oxidation decomposition/reduction potentials of organic pre‐sodiation materials are briefly introduced, with a focus on the structure‐activity relationship between functional group modifications and pre‐sodiation potential. Future challenges and directions for developing next‐generation organic pre‐sodiation technologies are also reviewed. The current review provides important insights into molecular engineering in organic pre‐sodiation, guiding the development of next‐generation technologies of SIBs.

## Graphical abstract

This review delves into the molecular engineering strategies for organic pre‐sodiation techniques, examining the intricate structure‐activity relationships between functional group modifications and oxidation decomposition/reduction potentials of organic pre‐sodiation materials. It also surveys recent breakthroughs in organic pre‐sodiation technology and identifies future research directions for molecular engineers to optimize performance and scalability in sodium‐ion batteries.

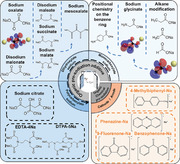

## 1 Introduction

**Figure 1:**
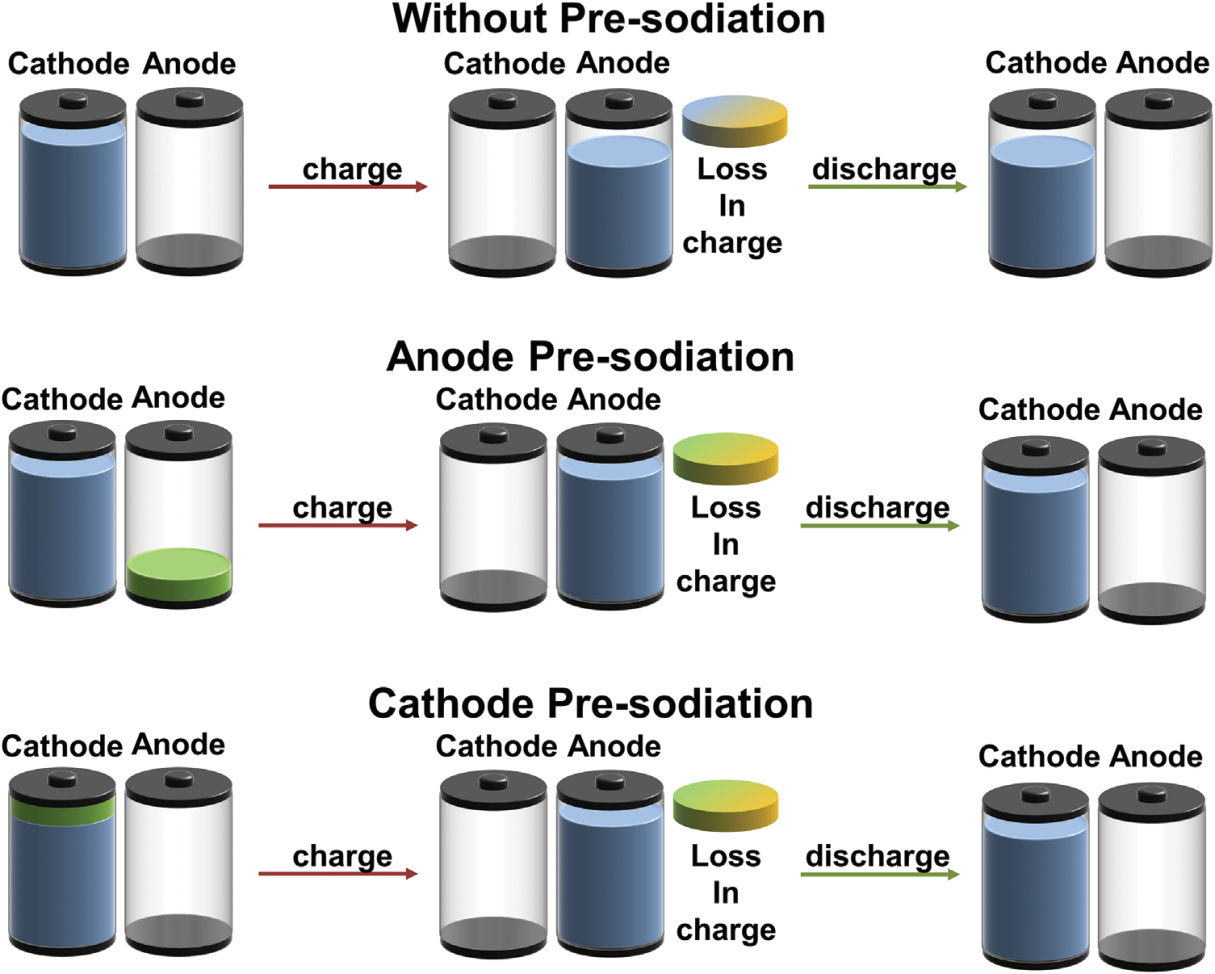
Scheme of pre‐sodiation technology.

The increasing demand for renewable energy sources has led to a surge in the adoption of electrochemical energy storage systems. Among these technologies, lithium‐ion batteries have garnered significant attention due to their high energy density, operating voltage, and cycle performance.^[^ ^1^ ^]^ However, concerns surrounding lithium scarcity, limited global distribution, and increased costs have sparked an exploration of alternative.^[^ ^2^ ^]^ Sodium‐ion batteries (SIBs), in particular, represent a promising avenue for advancing electrochemical energy storage systems, as sodium is more abundant and cost‐effective than lithium.^[^ ^3^, ^4^, ^5^ ^]^ Despite this potential, commercialization of sodium‐ion batteries faces significant challenges, primarily arising from three key factors: 1) the formation of solid electrolyte interphase layer during the initial cycle, which consumes up to 15% of active sodium ions;^[^ ^6^ ^]^ 2) side reactions at the anode that deplete active sodium ions, such as irreversible trapping in defects and functional groups or significant volume changes causing cracking; and 3) intrinsic sodium deficiency in commonly used cathode materials. To deal with these challenges, an excess of cathode material is often required in full cells, reducing material utilization efficiency and overall energy density. Recent advancements in pre‐sodiation technologies offer promising solutions to mitigate sodium deficiency and minimize sodium loss (**Figure** **1**).^[^ ^7^, ^8^ ^]^

Pre‐sodiation strategies can be classified into inorganic and organic methods. Inorganic approaches encompass electrochemical pre‐sodiation, direct contact with metallic sodium, and various inorganic additives. Electrochemical pre‐sodiation allows for precise control over the degree of pre‐sodiation by adjusting the discharge cutoff voltage. However, its complex disassembly process hinders large‐scale applications. Direct contact with metallic sodium involves combining the electrode material with metallic sodium using techniques such as vacuum thermal evaporation,^[^ ^9^ ^]^ stirring,^[^ ^10^ ^]^ or high‐energy ball milling,^[^ ^11^ ^]^ occasionally incorporating a small amount of electrolyte to facilitate contact.^[^ ^12^, ^13^, ^14^, ^15^, ^16^ ^]^ The potential difference between the electrode material and metallic sodium drives the pre‐sodiation process. Nevertheless, the high reactivity of metallic sodium necessitates that direct‐contact pre‐sodiation be performed in a glove box to prevent contamination and ensure consistent results. Furthermore, uneven contact can result in inconsistent pre‐sodiation, affecting battery capacity retention.^[^ ^14^ ^]^ Inorganic additives have been explored for pre‐sodiation purposes, including NaN_3_,^[^ ^17^, ^18^ ^]^ Na_2_O,^[^ ^19^, ^20^ ^]^ Na_3_P,^[^ ^21^ ^]^ and NaCrO_2_.^[^ ^22^ ^]^ However, their application is complicated by issues such as gas production, residual “dead mass,” toxicity, and explosion risk.

In contrast, organic pre‐sodiation techniques have garnered significant attention due to their abundant resources, structural diversity, and potential to overcome the limitations of traditional methods.^[^ ^23^ ^]^ Two primary approaches have emerged: organic pre‐sodiation additives and chemical pre‐sodiation. Organic pre‐sodiation additives are integrated into battery cathode slurry for pre‐sodiation, where they undergo irreversible decomposition at a specific oxidation voltage during the first charge cycle, releasing active sodium ions that facilitate pre‐sodiation. This process relies on precise control of the oxidative decomposition voltage, which must align with the operating potential of cathode materials and remain below the cut‐off charging voltage. Currently, reported organic pre‐sodiation additives primarily comprise sodium carboxylate salts and carbonated cyclic organic salts. While the latter, such as Na_4_C_6_O_6_, exhibits higher cost and raises concerns about the thermodynamic stability. Sodium carboxylates offer an attractive alternative, which can be readily synthesized from low‐cost, naturally derived organic acid precursors, rendering them an increasingly popular subject of research inquiry.^[^ ^24^, ^25^ ^]^ Chemical pre‐sodiation involves synthesizing polycyclic arylsodium compounds through reactions between metallic sodium and polycyclic aromatic hydrocarbons (PAHs) or other aromatic compounds in an organic solvent. These compounds are then applied to target electrode materials via spraying or immersion, enabling pre‐sodiation. The difference between the reduction potential of these compounds and the sodium intercalation potential of the electrode material facilitates rapid electrons and sodium ions transfer, ensuring efficient pre‐sodiation. Based on the discussion above, it is obvious that the effectiveness of both techniques hinges on precise control of electrochemical properties. For organic pre‐sodiation additives, the oxidative decomposition voltage is critical to ensure compatibility with standard sodium‐ion battery electrodes and maintain efficient decomposition to release active sodium ions. For chemical pre‐sodiation, the reduction potential of polycyclic arylsodium compounds must be carefully balanced with the sodium intercalation potential of the target electrode material to avoid incomplete or excessive pre‐sodiation. Therefore, precise pre‐sodiation potential control is essential for organic pre‐sodiation technology.

The molecular design of organic sodium carboxylates and polycyclic arylsodium compounds plays a pivotal role in regulating electrochemical potentials through precise modulation of their reactivity. By incorporating functional groups featuring specific atomic or cluster arrangements, researchers can leverage the unique electronegative and bonding properties of these moieties to modify the overall reactivity of organic molecules, thereby controlling reaction potential during pre‐sodiation processes. For instance, Hu et al.^[^ ^26^ ^]^ incorporated electron‐donating methyl groups into the molecular structure of sodium formate, which significantly lowered the decomposition voltage of modified sodium acetate from over 4 to 3.6 V, leading to a decomposition efficiency of up to 92.0% during the initial cycle. In a separate study, Man et al.^[^ ^27^ ^]^ utilized methyl‐modified 4‐methylbiphenyl for anode chemical pre‐sodiation. The exceptionally low reduction potential of 0.146 V facilitated the efficient intercalation of sodium ions into the anode due to the potential difference, achieving an initial Coulombic efficiency (ICE) of 99.1% for the pre‐sodiated hard carbon. Although numerous studies have explored molecular modification applications in this field, there still remains a dearth of comprehensive reviews that comprehensively summarize the fundamentals, principles, advances, and potential developmental directions of molecular engineering strategies for deep understanding and more rational design of efficient organic pre‐sodiation systems.

**Figure 2:**
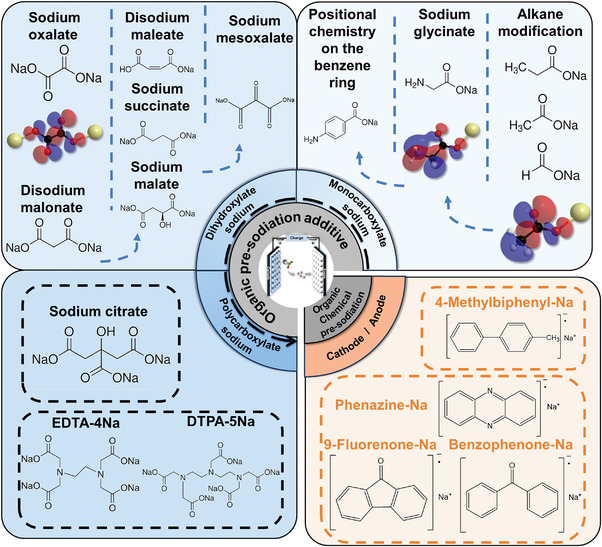
Molecular engineering strategies for organic pre‐sodiation.

This timely review aims to present the crucial role of molecular engineering in organic pre‐sodiation and provide an up‐to‐date overview of this field. After the introducing of the importance of molecular engineering strategies for efficient organic pre‐sodiation, fundamentals of organic sodium carboxylate additives and polycyclic arylsodium compounds for precisely regulating pre‐sodiation potential through substituent modifications are highlighted. By examining the mechanisms underlying these molecular engineering strategies, the practical performance of various organic sodium carboxylate additives and polycyclic arylsodium compounds developed through molecular engineering are reviewed (**Figure** **2**). Finally, future challenges and directions for molecular engineering in organic pre‐sodiation will be presented. The current review provides a comprehensive framework spanning theoretical foundations to practical examples of state‐of‐the‐art molecular engineering strategies for pre‐sodiation could inspire the development of efficient and effective organic pre‐sodiation systems.

## 2 Fundamentals of Molecular Engineering Strategies for Organic Pre‐Sodiation

Organic additives and chemical pre‐sodiation are two burgeoning approaches to organic pre‐sodiation, which exhibit considerable promise for large‐scale applications due to the versatility of organic materials and the vast array of synthesis methodologies available. In this part, we will provide a concise overview of the fundamental principles underlying these two techniques, followed by an in‐depth examination of molecular engineering strategies for optimizing the potential of organic pre‐sodiation.

Sodium carboxylate additives have garnered attention due to the unique properties of carbon atoms and their capacity for diverse structural designs through combination with other elements. However, the application of carboxylate salts as pre‐sodiation additives in sodium‐ion batteries is hindered by their inherent limitations. Specifically, the oxidative decomposition voltage of monocarboxylate salts such as sodium formate and dicarboxylate salts like sodium oxalate can be prohibitively high, reaching 4.19 V (vs Na/Na^+^, the voltages mentioned in the following text are all relative to Na/Na^+^, unless otherwise noted) for sodium formate and 4.41 V for sodium oxalate.^[^ ^28^, ^29^ ^]^ This excessive voltage increase can lead to electrolyte decomposition and other detrimental side reactions, thereby diminishing the pre‐sodiation effectiveness of these additives.^[^ ^30^ ^]^ Therefore, it is critical to minimize the oxidative decomposition potential of carboxylate sodium additives during the pre‐sodiation process to ensure the successful incorporation of active sodium ions into battery systems.

Chemical pre‐sodiation has garnered significant attention in recent years due to its advantages of simplicity, uniform reaction, and controllability. Notably, the driving force for the pre‐sodiation process lies in the potential difference between the polycyclic arylsodium compounds and the target materials to be pre‐sodiated. To optimize the effectiveness of chemical pre‐sodiation, it is essential to precisely control the reduction potential of these compounds, thereby regulating the entire process. Unlike sodium carboxylate additives, which primarily seek to minimize the oxidative decomposition potential, regulating the reduction potential of polycyclic arylsodium compounds necessitates consideration of the specific characteristics of different electrodes to be sodiated, adhering to the “redox potential matching principle”.^[^ ^31^ ^]^ When chemically pre‐sodiating anodes, the reduction potential of polycyclic arylsodium compounds must be lower than the sodium‐ion insertion potential of the electrode to ensure effective supplementation of active sodium ions. For cathode pre‐sodiation, the optimal range for the reduction potential is crucial, as over‐sodiation can lead to irreversible crystal structure collapse or interface damage, negatively impacting electrochemical performance.^[^ ^32^, ^33^, ^34^ ^]^

**Figure 3:**
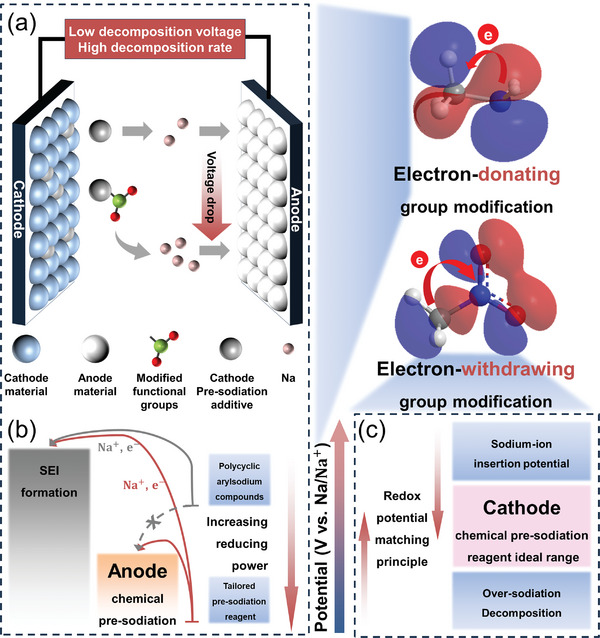
a) Schematic of molecular engineering strategies in sodium carboxylate additives. b) Schematic of molecular engineering strategies in anode chemical pre‐sodiation. c) Schematic of molecular engineering strategies in cathode chemical pre‐sodiation.

To mitigate the issue of high decomposition voltage in sodium carboxylate additives and to modulate the reducing ability of polycyclic arylsodium compounds in chemical pre‐sodiation, researchers have explored various strategies, including size control, conductive carbon coupling, catalyst introduction, and molecular structure modification. While simple conductive carbon coupling and size regulation can be effective to some extent, they often fall short of achieving a suitable potential. The incorporation of catalysts into the battery system may introduce new substances that could compromise its performance, particularly in complex electrochemical environments. A recent study has demonstrated the importance of considering the interactions between different catalysts and solvents in Li‐S battery systems, revealing the potential detrimental effects of these catalysts on electrolyte stability.^[^ ^35^ ^]^ In contrast, modifying the functional groups of these organic compounds offers an effective strategy for fine‐tuning the energy level of the Highest Occupied Molecular Orbital (HOMO), thereby influencing their redox potential. The HOMO energy is primarily dependent on the electron density and π‐electron delocalization across a π‐conjugated backbone. Electron‐donating substituents, such as those with lone pair donors or alkyl groups, can raise the HOMO energy, reducing the solid‐state ionization potential. Conversely, electron‐withdrawing groups like ‐F and ‐C≡N lower both HOMO and Lowest Unoccupied Molecular Orbital energies, increasing the solid‐state electron affinity.^[^ ^36^ ^]^ According to Koopman's theorem, the ionization potential of a molecule can be approximated as the negative value of its HOMO energy.^[^ ^37^, ^38^ ^]^ For sodium carboxylate additive, a higher HOMO energy level generally corresponds to a lower ionization potential, indicating that electrons are more easily removed during oxidation. This suggests that introducing electron‐donating functional groups into sodium carboxylate additives can weaken the oxygen‐sodium bond, thereby lowering the oxidation potential and optimizing the electrochemical performance (**Figure** **3**a). For chemical pre‐sodiation, a lower HOMO energy level corresponds to a higher reduction potential, indicating that aromatic anionic radicals have greater difficulty providing electrons. Conversely, a higher HOMO energy level is associated with a lower reduction potential, facilitating easier electron transfer. Therefore, for anode chemical pre‐sodiation, it is necessary to introduce electron‐donating functional groups into polycyclic arylsodium compounds to reduce the reduction potential below the sodium‐ion intercalation potential of the target electrode (Figure 3b). In contrast, appropriate electron‐withdrawing group modifications are generally suitable for cathode chemical pre‐sodiation to create a more moderate reduction environment (Figure 3c).

The molecular engineering approach of organic pre‐sodiation enables precise control over the reaction potential by strategically introducing functional groups into sodium carboxylate additives and polycyclic arylsodium compounds. This strategy leverages a fundamental understanding of the relationships between chemical structure, redox properties, and sodiation kinetics to optimize the performance of organic pre‐sodiation techniques. In the following sections, we will critically review recent advances in organic pre‐sodiation, highlighting the crucial roles of functional group modifications on reaction potential and sodiation efficiency.

## 3 Organic Pre‐Sodiation Technologies for Sodium‐Ion Batteries

### 3.1 Organic Sodium Carboxylate Additives

Organic sodium carboxylates, including monocarboxylates (e.g., sodium formate) and dihydroxylates (e.g., sodium oxalate), have emerged as a promising pre‐sodiation additives due to their abundance, affordability, and eco‐friendly characteristics. However, their sodium‐ion release capacity is often limited by the high oxidative decomposition voltages, which can compromise battery performance. To deal with this issue, molecular engineering though functional group modifications are introduced to reduce the oxidative decomposition voltage of organic sodium carboxylates, thereby enhancing their pre‐sodiation efficiency.

#### 3.1.1 Monocarboxylic Sodium Additives

**Figure 4:**
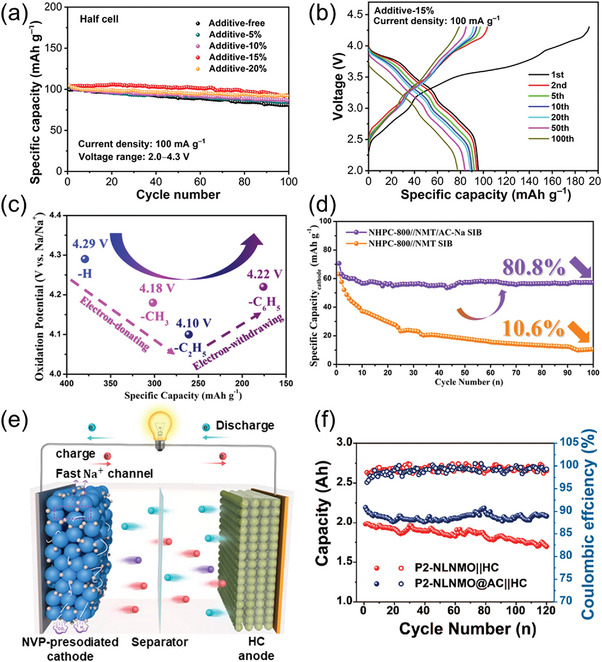
a) Cycling performance of NNZMO with different sodium formate content at 100 mA g^−1^ for a half cell. b) Charge‐discharge curves of the HC||NNZMO‐15% sodium formate full cell at 100 mA g^−1^ (calculated based on the active material of the cathode).^[^ ^28^ ^]^ Copyright 2024, Springer Nature. c) Comparison of oxidation potentials of four mono‐sodium carboxylates. d) Comparison of the cycling stability of NHPC//NMT and NHPC//NMT/AC‐Na SIB at 1 A g^−1^.^[^ ^39^ ^]^ Copyright 2021, Wiley‐VCH. e) Schematic diagram of the NVP‐10% Ac‐Na || HC full cell.^[^ ^26^ ^]^ Copyright 2024, American Chemical Society. f) Cycling performance of P2‐NLNMO||HC and P2‐NLNMO@AC‐Na||HC pouch cells at 0.3 C.^[^ ^40^ ^]^ Copyright 2023, American Chemical Society.

In the context of SIBs, monocarboxylic sodium compounds are characterized by the presence of a single sodium carboxylate group. Notably, sodium formate, the simplest representative of this class, has been found to possess an impressive theoretical specific capacity of 394 mAh g^−1^, underscoring its potential as a pre‐sodiation additive in SIBs applications. Its non‐toxic nature, widespread availability, and low cost make it an attractive candidate for high‐performance pre‐sodiation. Zhao et al.^[^ ^28^ ^]^ demonstrated the potential of sodium formate as a pre‐sodiation additive in P2‐type manganese‐based layered oxide (Na_0.66_Ni_0.26_Zn_0.07_Mn_0.67_O_2_, NNZMO). In half‐cell tests, the addition of sodium formate to the NNZMO cathode resulted in enhanced capacity retention compared to the unmodified cathode (**Figure** **4**a). A full cell comprising a NNZMO cathode with 15 wt.% HCOONa as a sodium compensation additive and a hard carbon (HC) anode delivered an initial discharge specific capacity of 95.7 mAh g^−1^ at a current density of 100 mA g^−1^, retaining 81.3% of the capacity after 100 cycles. This performance outperformed that of the unmodified NNZMO cathode, which exhibited a specific capacity of 71.8 mAh g^−1^ and 55.7% retention. (Figure 4b). However, electrochemical decomposition tests revealed that sodium formate displayed a charge‐specific capacity of only 295.3 mAh g^−1^ when charged to 4.5 V during the first cycle, substantially lower than its theoretical specific capacity.^[^ ^39^ ^]^ This finding highlights the importance of considering the actual pre‐sodiation efficiency in addition to theoretical predictions. The molecular engineering strategy of introducing electron‐donating functional groups to modify organic molecules and elevate their HOMO energy levels effectively reduces the oxidative decomposition voltage of sodium carboxylates. To deal with this limitation, researchers have successfully utilized molecular engineering strategies by introducing electron‐donating functional groups to modify HOMO energy levels of sodium formate, demonstrating a significant reduction in decomposition voltage (**Table** **1**).

**Table 1:** Pre‐sodiation properties of various monocarboxylate sodium additives.

Materials	Substituent	HOMO Energy/eV	Oxidation Potential based on GCD curve/V	First Charge specific capacity/mAh g^−1^	Available Content/% Na^+^ (Charge cut‐off Voltage)	Refs.
sodium formate (FM‐Na)	‐H	∖	4.29	379.3	96.26 (4.5 V)	[39]
			4.19	295.3	74.94 (4.5 V)	[28]
sodium benzoate (BZ‐Na)	‐C_6_H_5_	−0.19033	4.22	175.9	94.56 (4.5 V)	[28]
sodium acetate (AC‐Na)	‐CH_3_	∖	4.18	301.8	92.57 (4.5 V)	[39]
			3.6	323/21	99 (4.2 V)	[26]
			≈4.1	≈300	92 (4.3 V)	[40]
sodium propionate (PP‐Na)	‐CH_2_CH_3_	−0.18192	4.1	261	93.54 (4.5 V)	[39]
sodium glycinate (GC‐Na)	‐CH_2_NH_2_	−0.17006	3.85	256.1	92.80 (4.5 V)	[41]

Alkyl groups, such as methyl and ethyl, which are commonly recognized as electron‐donating groups and could increase the electron density of carboxylic groups, have been shown to weaken the O‐Na bond strength, thereby lowering the oxidation potential of sodium carboxylates. For instance, Zou et al.^[^ ^39^ ^]^ quantitatively investigated this effect using DFT calculations and experimental testing, revealing that substituting hydrogen atoms with electron‐donating alkyl groups decreases the oxidative decomposition voltage of sodium carboxylates. As shown in Figure 4c, Galvanostatic charge/discharge (GCD) curve analysis revealed that the oxidative decomposition voltages of sodium acetate (AC‐Na) and sodium propionate were 4.18 and 4.10 V, respectively. As a pre‐sodiation additive, sodium acetate has been demonstrated to enhance its effectiveness by providing an additional charge capacity of 100.1 mAh g^−1^ during the first charging cycle when coupled with self‐synthesized sodium‐deficient P2‐Na_2/3_Ni_1/3_Mn_2/3_₋_x_Ti_x_O_2_ (x = 1/10, NMT) layered oxide cathode in a half cell system. Furthermore, the full cell comprising sodium acetate, NMT, and 3D nitrogen‐doped hierarchical porous carbon (NHPC) exhibited improved cycle stability, retaining 80.8% of its initial capacity after 100 cycles (Figure 4d). Hu et al.^[^ ^26^ ^]^ demonstrated that when employed as a pre‐sodiation additive to the Na_3_V_2_(PO_4_)_3_ (NVP) cathode at a concentration of 10%, Ac‐Na decomposed at low voltage, releasing active sodium ions and generating gas that created a porous structure in the cathode (Figure 4e). This structure was found to reduce polarization overpotential under high load, improve rate performance, and significantly increase battery energy density by eliminating residual “dead mass”. Furthermore, Zhang et al.^[^ ^40^ ^]^ have innovatively transformed residual alkali in the layered oxide cathode into sodium acetate, which exhibited a high pre‐sodiation specific capacity of 326 mAh g^−1^ and a low decomposition voltage of 4.1 V, leading to increased energy density of the P2‐Na_0.85_Li_0.12_Ni_0.22_Mn_0.66_O_2_ (P2‐NLNMO)@AC‐Na||HC full cell of 130 Wh kg^−1^ while maintaining a capacity retention rate of 95.1% over 120 cycles (Figure 4f). Based on the discussion above, it can be concluded that the decomposition potential of sodium acetate is significantly lower than that of other pre‐sodiation additives, rendering it an effective and practical choice for enhancing the performance of sodium‐ion batteries.

**Figure 5:**
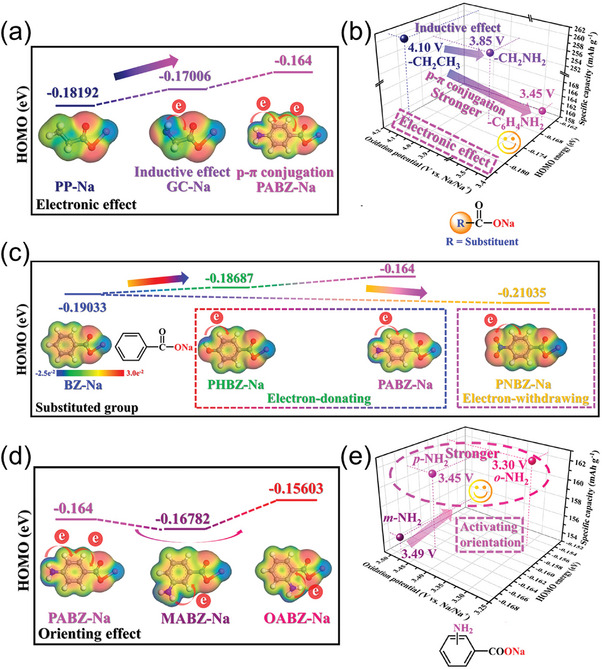
a) HOMO energy of three sodium carboxylates with different electronic effects, calculated using the DFT method. b) Comparison of the oxidation potentials of PP‐Na, GC‐Na, and PABZ electrodes. c) HOMO energies were calculated using the DFT method of BZ‐Na and three BZ‐Na derivatives with different para‐substituted functional groups. d) HOMO energy of three ABZ‐Na isomers, calculated using the DFT method. e) Comparison of the oxidation potentials of three ABZ‐Na isomers.^[^ ^39^ ^]^ Copyright 2021, Wiley‐VCH.

In addition, the amino group's enhanced electron‐donating ability, arising from the nitrogen atom's lone pair electrons capable of delocalization, could also significantly modulate the decomposition potential of sodium carboxylates.  Zou et al.^[^ ^41^ ^]^ investigated the regiochemical effects of NH_2_ groups on glycine sodium (GC‐Na) and its influence on electron‐deficient aromatic systems (**Table** **2**). DFT calculations revealed that the amino group's inductive effect increases the HOMO energy of GC‐Na to ‐0.18192 eV, which is higher than that of ethyl‐modified sodium propionate, indicating a significant reduction in its decomposition potential to 3.85 V. Furthermore, the introduction of an amino group onto the benzene ring allows for resonance and inductive effects, further enhancing the electron cloud density and raising the HOMO energy of sodium para‐aminobenzoate to −0.164 eV, thereby lowering its oxidative decomposition potential to 3.45 V (**Figure** **5**a,b). Meanwhile, Zou et al.^[^ ^41^ ^]^ conducted a comparative analysis examining the potential regulatory capabilities of nitro and hydroxyl groups in aromatic ring systems. Their findings revealed that the nitro group's strong electron‐withdrawing effect consistently shifts electron density away from the aromatic framework, lowering the HOMO energy of the corresponding sodium carboxylate.^[^ ^41^ ^]^ In contrast, the pre‐sodiation characteristics of the hydroxyl group resemble those of the amino group; however, due to oxygen's higher electronegativity (3.44) compared to nitrogen (3.04), its ability to donate electrons is limited. Consequently, the increase in HOMO energy and reduction in decomposition potential is less pronounced for the hydroxyl group than for the amino group (Figure 5c).

**Table 2:** Pre‐sodiation properties of various monocarboxylate sodium additives (Modification of benzene ring).

Materials	Substituent	HOMO energy/eV	Oxidation potential based on GCD curve/V	First Charge specific capacity/mAh g^−1^	Available content/% Na^+^ (Charge cut‐off voltage)	Refs.
Sodium p‐nitrobenzoate (PNBZ‐Na)	*p‐*NO_2_	−0.21035	∖	36.5	25.90% (4.5 V)	[41]
sodium benzoate (BZ‐Na)	‐C_6_H_5_	−0.19033	4.22	175.9	94.56% (4.5 V)	[41]
sodium p‐hydroxybenzoate (PHBZ‐Na)	*p‐*OH	−0.18687	3.55	156.2	93.50% (4.5 V)	[41]
sodium p‐aminobenzoate (PABZ‐Na)	*p‐*NH_2_	−0.164	3.45	161.1	95.90% (4.5 V)	[41]
sodium m‐aminobenzoate (MABZ‐Na)	*m‐*NH_2_	−0.16782	3.49	154.8	92.10% (4.5 V)	[41]
sodium p‐aminobenzoate (PABZ‐Na)	*p‐*NH_2_	−0.164	3.45	161.1	95.90% (4.5 V)	[41]
sodium o‐aminobenzoate (OABZ‐Na)	*o‐*NH_2_	−0.15603	3.3	162.3	96.60% (4.5 V)	[41]

Additionally, Zou et al.^[^ ^41^ ^]^ conducted an in‐depth investigation into the positional chemistry of modified functional groups within aromatic ring systems (Table 2). They analyzed the regioselectivity of amino group substitution on aromatic systems by evaluating the pre‐sodiation performance of three isomers formed by amino modifications at the para, meta, and ortho positions of the benzene ring. The results indicated that the introduction of electron‐donating groups at the ortho‐ and para‐positions in the aromatic ring system activates the ring through resonance effects, whereas meta‐substitution does not exhibit this level of activation. Moreover, these electron‐donating group modifications at the ortho‐ and para‐positions direct the phenyl carbon atoms attached to the ‐COONa group, thereby increasing the electron density. As a consequence, ortho‐ and para‐functional groups significantly elevate the HOMO energy level, leading to a substantial reduction in the oxidation potential of sodium carboxylate (Figure 5d,e). Notably, the ortho‐substituent exhibits a more pronounced electron‐donating effect, resulting in a lower oxidation decomposition voltage for the ortho‐isomer compared to the para‐isomer. The positional chemical trend is also observed in the benzene ring system with hydroxy and nitro groups. As electron‐donating groups, the regioisomers of hydroxy benzoate sodium exhibit similar pre‐sodiation characteristics to amino benzoate sodium. In contrast, the electron‐withdrawing nitro modification reduces the electron density of the aromatic ring's core structure, lowers the HOMO energy level, and increases the oxidation decomposition voltage.

Based on the advances discussed above, the incorporation of electron‐donating groups, such as alkyl or amino moieties, into the molecular framework of sodium formate‐based additives has been demonstrated to effectively reduce the oxidative decomposition voltage of monocarboxylate species. This phenomenon can be attributed to the enhanced electron donation capabilities of these modifying groups. Notably, the size and characteristics of these substituents exert a profound influence on the overall performance of the molecular additive. As such, it is essential to strike a balance between inductive effects and steric hindrance when designing bulky substituent modifications, thereby optimizing the development of high‐performance monocarboxylate sodium additives. This nuanced understanding paves the way for future research directions in this area, ultimately informing the design of more effective pre‐sodiation strategies for SIBs.

#### 3.1.2 Dihydroxylate Sodium Additives

The modification of sodium monocarboxylate salts through the introduction of effective electron‐donating groups has been shown to significantly increase the HOMO energy level of sodium formate, thereby reducing its oxidative decomposition voltage and facilitating the release of active sodium ions. This molecular engineering strategy can be applied to dihydroxylate sodium additives as well (**Table** **3**). Sodium oxalate (Na_2_C_2_O_4_), a representative of sodium dicarboxylate salts, has been identified as an ideal pre‐sodiation additive due to its high theoretical specific capacity (400 mAh g^−1^), low cost, and good air stability.^[^ ^42^ ^]^ However, the actual decomposition voltage always exceeds its theoretical value, leading to excessive activation potential and then limiting the suitability for use with common layered oxide or phosphate cathodes.^[^ ^43^, ^44^ ^]^ To overcome these limitations, molecular engineering strategies have been developed by incorporating electron‐donating groups into the structure of sodium oxalate to enhance the HOMO energy level, thereby reducing the oxidative decomposition voltage.  In contrast to conventional modification methods, such as catalyst composites,^[^ ^45^, ^46^ ^]^ intricate conductive network construction,^[^ ^47^ ^]^ and nanoscale particle size refinement,^[^ ^42^, ^48^ ^]^ which face challenges in terms of cost‐effectiveness, processing complexity, and industrial scalability, molecular engineering strategy offers a more straightforward and effective approach.

**Table 3:** Pre‐sodiation properties of various dihydroxylate sodium additives.

Materials	Substituent	HOMO energy/eV	Oxidation potential based on GCD curve/V	First Charge specific capacity/mAh g^−1^	Available content/% Na^+^ (Charge cut‐off voltage)	Refs.
disodium terephthalate (TP‐2Na)	‐C_6_H_4_‐	∖	none	25.2	9.88 (4.5 V)	[39]
disodium maleate (MA‐2Na)	‐CH=CH‐	∖	4.36	104.4	31.16 (4.5 V)	[39]
sodium oxalate (OA‐2Na)	none	−0.25	4.19	376.8	94.20 (4.5 V)	[39, 47]
sodium succinate(SS)	‐CH_2_CH_2_‐	∖	4.05	325.8	98.72 (4.5 V)	[49]
sodium mesoxalate (Na_2_C_3_O_5_)	‐CO‐	∖	4	310	94 (4.2 V)	[50]
disodium malonate(MN‐2Na)	‐CH_2_‐	−0.24	3.98	124.3	34.33 (4.5 V)	[39, 47]
sodium malate (SM)	‐CH(OH)CH_2_‐	∖	3.85	289.5	96.50 (4.5 V)	[49]

**Figure 6:**
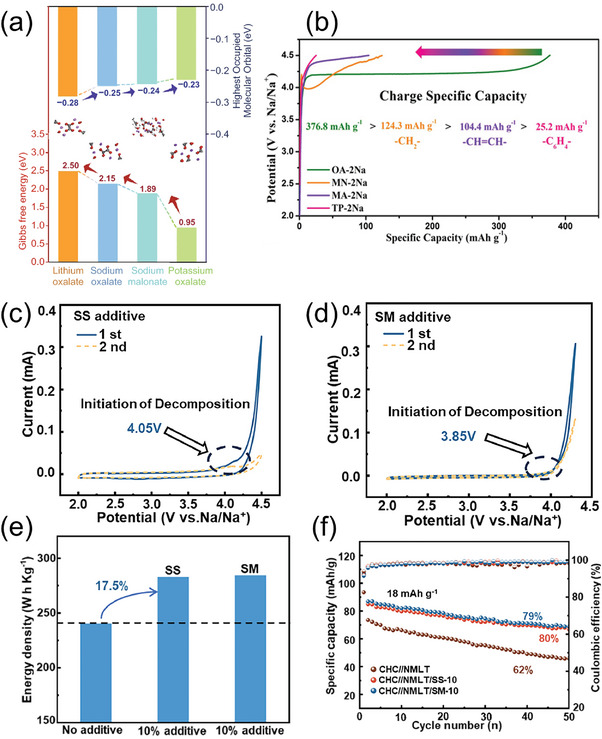
a) HOMO level of sodium malonate (inset shows the corresponding molecular structure).^[^ ^47^ ^]^ Copyright 2022, Springer Nature. b) Galvanostatic charge profiles of four disodium carboxylates at 0.1 A g^−1^.^[^ ^39^ ^]^ Copyright 2021, Wiley‐VCH. c) CV curves at a scan rate of 0.1 mV s^−1^ for the SS electrode. d) CV curves at a scan rate of 0.1 mV s^−1^ for the SM electrode. Comparison of electrochemical performance of the hard carbon/Na_0.78_Ni_0.2_Mn_0.7_Li_0.05_Ti_0.05_O_2_ full cells without or with 10 wt.% SS/SM additives. e) Energy density comparison based on the total mass of the anode and cathode active materials. f) Cycling performance at 0.1C.^[^ ^49^ ^]^ Copyright 2024, Elsevier.

The introduction of electron‐donating alkyl groups into dihydroxylate sodium additives has been shown to effectively lower their decomposition voltage. Song et al.^[^ ^47^ ^]^ investigated the oxidative decomposition potential of sodium malonate by introducing a methylene bridge between two sodium carboxylate molecular clusters using DFT calculations and experimental methods. Their results demonstrated that this modification elevated the HOMO energy level of sodium malonate to −0.24 eV (**Figure** **6**a), weakening the interaction between carboxylate ions and sodium ions. Cyclic voltammetry tests revealed the reduced oxidative decomposition potential of sodium malonate, indicating it could be a suitable pre‐sodiation additive for sodium‐based electrochemical energy storage systems. Similarly, Zou et al.^[^ ^39^ ^]^ further explored the pre‐sodiation characteristics of sodium malonate during the first charge process. At a current of 0.1 A g^−1^, the first charge curve revealed that sodium malonate decomposed at 3.98 V and continued to supply active sodium ions to the battery system during subsequent charging processes (Figure 6b). These findings substantiate the effectiveness of incorporating electron‐donating groups into dihydroxylate sodium additives for improved pre‐sodiation performance.

In addition, in contrast to electron‐donating alkyl groups that significantly lower the oxidative decomposition voltage of sodium carboxylate additives, the incorporation of electron‐withdrawing groups with conjugated properties, such as carbon‐carbon double bonds or benzene rings, into the molecular structure of sodium dicarboxylate salts strengthens the O‐Na bond. Consequently, the oxidative decomposition voltage increases. For instance, corroborated this finding by demonstrating that the presence of a carbon‐carbon double bond in disodium maleate significantly elevated its decomposition potential to 4.36 V (Figure 6b).^[^ ^39^ ^]^ To mitigate the adverse effects of conjugated systems on the decomposition potential of sodium carboxylates, Yang et al.^[^ ^49^ ^]^ employed a dual strategy that combined structural modification to disrupt the carbon‐carbon double bond with functional group modification by introducing hydroxyl groups. This approach significantly reduced the oxidative decomposition potential of dicarboxylates. Disrupting the carbon‐carbon double bond in disodium maleate enhanced the electron cloud density in the sodium succinate molecular chain, lowering the decomposition potential to 4.05 V (Figure 6c). And the introduction of electron‐donating hydroxyl groups into sodium succinate further increased the electron cloud density along the main molecular chain of sodium malate. Consequently, the oxidative decomposition potential of sodium malate decreased further to 3.85 V (Figure 6d). This innovative molecular design enabled sodium succinate and sodium malate to achieve excellent pre‐sodiation performance, with utilization rates exceeding 95% of their theoretical capacities. The addition of 10 wt.% sodium succinate or sodium malate increased the capacity of the hard carbon/Na_0.78_Ni_0.2_Mn_0.7_Li_0.05_Ti_0.05_O_2_ full cell from 240 Wh kg^−1^ to 282 Wh kg^−1^ and 284 Wh kg^−1^, respectively (Figure 6e). Moreover, after 50 cycles, the capacity retention of the full cell improved from 62% to 80% and 79%, respectively, significantly enhancing cycling stability (Figure 6f).

**Figure 7:**
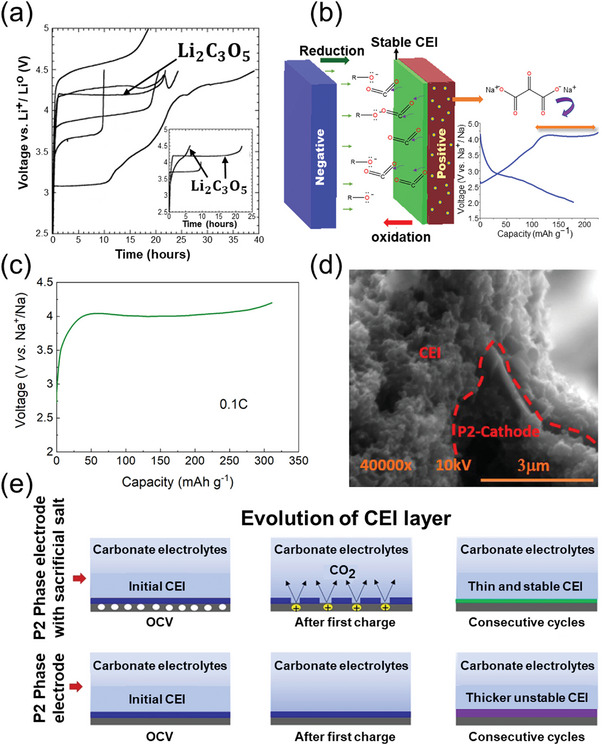
a) Charge curve of Li_2_C_3_O_5_ (inset: charge curve of Li_2_C_3_O_5_ after compositing with carbon material).^[^ ^51^ ^]^ Copyright 2010, Elsevier. b) Schematic of the Na_2_C_3_O_5_ decomposition. c) Galvanostatic oxidation profile of Na_2_C_3_O_5_ + KJ600 (70:30 wt.%) sacrificial sodium salt at a C‐rate of 0.1C. d) Cross‐sectional analysis of electrodes containing sacrificial sodium salt after 10 cycles. e) Schematic of the evolution of CEI layer for P2‐Na_2/3_Mn_0.8_Fe_0.1_Ti_0.1_O_2_ with sacrificial sodium salt (top) and P2‐Na_2/3_Mn_0.8_Fe_0.1_Ti_0.1_O_2_ (bottom).^[^ ^50^ ^]^ Copyright 2021, American Chemical Society.

Moreover, Shanmukaraj et al.^[^ ^51^ ^]^ explored the employment of higher homologues in the oxalate family as pre‐sodiation additives. Their findings revealed that the high density of the HOMO state in ketomalonate [(CO_2_)_2_CO]^2−^ facilitates electron detachment, thereby enhancing the oxidative decomposition of corresponding metal salt molecules. The electrochemical charging performance of di‐lithium ketomalonate (Li_2_C_3_O_5_) was evaluated using electrodes comprising a 30% KJ‐600 conductive carbon blend. Notably, the first‐cycle charging curve exhibits an oxidative decomposition voltage of ≈4.2 V (**Figure** **7**a), which is lower than that observed for lithium oxalate. This finding provides indirect evidence that a higher electron state density positively influences the decomposition voltage of polycarboxylate sodium salts. Furthermore, mesoxalate, a biooxidation product of glycerol, offers advantages in terms of low cost and broad availability, rendering it a promising candidate for large‐scale pre‐sodiation applications. Inspired by the functional group‐modified structure of Li_2_C_3_O_5_, Fernández‐Ropero et al.^[^ ^50^ ^]^ employed carbonyl‐modified sodium mesoxalate (Na_2_C_3_O_5_) as a pre‐sodiation additive for P2‐Na_2/3_Mn_0.8_Fe_0.1_Ti_0.1_O_2_ sodium‐ion cathodes (Figure 7b). The low decomposition voltage of Na_2_C_3_O_5_ at 4.0 V facilitated efficient decomposition of the pre‐sodiation additive, yielding a specific decomposition capacity of ≈310 mAh g^−1^ at 0.1 C, with a decomposition efficiency of up to 94% (Figure 7c). Sufficient release of active sodium ions from the additive enhanced the specific capacity of the half‐cell by 27% (164 mAh g_P2_^−1^), and full‐cell studies demonstrated a fourfold increase in specific capacity with hard carbon anodes. Furthermore, scanning electron microscopy (Figure 7d) and X‐ray photoelectron spectroscopy analyses revealed that the introduction of Na_2_C_3_O_5_ as a sacrificial sodium salt and the release of CO_2_ gas during the decomposition process promoted the formation of a thin and stable cathode electrolyte interphase (CEI) layer on the anode surface of the sodium‐ion battery (Figure 7e).

In summary, the molecular engineering strategy of the dihydroxylate sodium additive is similar to that of the monocarboxylic sodium additive. By incorporating electron‐donating groups into the molecular structure of sodium oxalate, the HOMO energy level is elevated, which reduces the oxidative decomposition voltage. Notably, the optimization of dihydroxylate sodium additives requires a comprehensive evaluation of multiple factors, including electronic effects, molecular stability, and reaction kinetics. These interdependent factors often involve trade‐offs, resulting in a complex multidimensional challenge in optimizing the pre‐sodiation performance of additives. For instance, while the introduction of electron‐donating alkyl groups into sodium malonate successfully reduced the decomposition voltage, DFT calculations reveal that sodium malonate exhibits a larger bandgap compared to sodium oxalate, indicating lower electron conductivity.^[^ ^47^ ^]^ This example illustrates the complexities inherent in molecular engineering strategies, where optimizations in one parameter may be compromised by unforeseen effects on others. To successfully develop dihydroxylate sodium additives with optimal pre‐sodiation performance, it is essential to consider multiple factors simultaneously and strike a balance between competing influences. By acknowledging these intricacies, researchers can design more effective molecular structures that maximize the benefits of pre‐sodiation in SIBs.

#### 3.1.3 Polycarboxylate Sodium Additives

For sodium carboxylate additives, both oxidative decomposition potential and effective sodium replenishment capacity are crucial factors influencing pre‐sodiation performance. The incorporation of electron‐donating groups, such as alkyl or hydroxyl moieties, into the molecular structure of dihydroxylate sodium additives can enhance the HOMO energy level and reduce the oxidative decomposition voltage. However, these modification groups often fail to provide active sodium ions, thereby hindering sodium replenishment capacity of additives. Zou et al.^[^ ^39^ ^]^ investigated the sodium supplementation capacity of sodium oxalate and its functionalized derivatives, revealing that aside from sodium oxalate, derivatives such as sodium malonate and sodium maleate exhibited relatively low initial charge capacities (<150 mAh g^−1^) (Figure 6c). This result suggests that these functionalized derivatives undergo intramolecular cyclization during the Kolbe electrolysis process, forming stable cyclic compounds that impede complete decomposition. Consequently, molecular engineering strategies must balance sodium replenishment capacity and oxidative decomposition voltage to optimize pre‐sodiation performance. The development of sodium carboxylate additives with multiple sodium carboxylate groups (>2) within a single molecule while maintaining a low oxidative decomposition potential is essential for achieving efficient sodium ions supplementation (**Table** **4**).

**Table 4:** Pre‐sodiation properties of various polycarboxylate sodium additives.

Materials	Substituent	HOMO energy/eV	Oxidation potential based on GCD curve/V	First Charge specific capacity/mAh g^−1^	Available content/% Na^+^ (Charge cut‐off voltage)	Refs.
sodium citrate (SC)	‐CH_2_‐C(OH)‐CH_2_‐	∖	≈4	302	97 (4.4 V)	[52]
ethylenediaminetetraacetic acid tetrasodium (EDTA‐4Na)	‐N‐CH_2_CH_2_‐N‐	∖	≈4	420	177.2 (4.5 V)	[53]
diethylenetriamine penta‐acetic acid pentasodium (DTPA‐5Na)	‐N‐CH_2_CH_2_‐N‐CH_2_CH_2_‐N‐	∖	≈4	363	136.38 (4.3 V)	[54]

**Figure 8:**
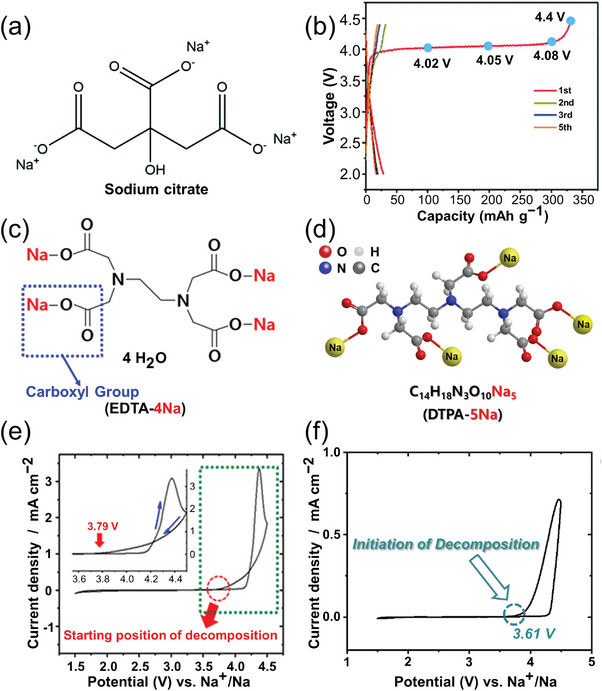
a) Chemical structural formula of SC. b) Galvanostatic charge/discharge curves of the SC electrode at 20 mA g^−1^.^[^ ^52^ ^]^ Copyright 2021, Royal Society of Chemistry. c) Chemical structural formula of EDTA‐4Na.^[^ ^53^ ^]^ Copyright 2019, American Chemical Society. d) Scheme showing multiple roles of DTPA‐5Na.^[^ ^54^ ^]^ Copyright 2020, Elsevier. e) CV in the potential range 2.0–4.5 V with a scan rate of 0.5 mV s^−1^ of the EDTA‐4Na electrode (inset: magnification of CV in the voltage range 3.6–4.5 V).^[^ ^53^ ^]^ Copyright 2019, American Chemical Society. f) CV curves in the potential range of 1.5–4.3 V at a scan rate of 0.1 mV s^−1^ for the DTPA‐5Na electrode. ^[^ ^54^ ^]^ Copyright 2020, Elsevier.

Sodium citrate, a tricarboxylic compound, contains three sodium carboxylate groups (‐COONa) and an electron‐donating hydroxyl group (‐OH), enables its unique chemical properties (**Figure** **8**a). The sodium carboxylate groups serve as effective sites for sodium ion exchange, enhancing the compound's sodium compensation capacity. In addition, the hydroxyl group and long alkyl chains, acting as electron donors, elevate the energy level of the HOMO, thereby lowering the oxidative decomposition voltage. This molecular engineering approach yields an efficient pre‐sodiation additive with increased active sodium sources and reduced decomposition voltages. As reported by Zhang et al.^[^ ^52^ ^]^, sodium citrate was first employed as a self‐compensating sodium additive for sodium‐ion batteries, exhibiting moderate oxidation at ≈4 V and delivering a practical sodiation capacity of 302 mAh g^−1^ (Figure 8b). When 10% sodium citrate was added to the NVPOF/rGO cathode and paired with an HC anode, the assembled full cell exhibited a significant 28.9% increase in energy density, underscoring its potential as a valuable pre‐sodiation additive for enhancing the performance of sodium‐ion batteries.

Researchers have developed a range of nitrogen‐containing chelating agents, which represent a sophisticated class of organic compounds based on the polycarboxylate sodium principle. The nitrogen framework of these agents enables the clustering of multiple carboxylate groups within a single molecular structure, thereby creating a more complex and effective binding environment. By incorporating these chelating agents as pre‐sodiation additives, researchers can introduce additional active sodium sources into battery systems, thereby enhancing the overall performance and lifespan of SIBs. Specifically, Ethylenediaminetetraacetic acid tetrasodium salt (EDTA‐4Na) and diethylenetriamine penta‐acetic acid salt (DTPA‐5Na) have been shown to facilitate the effective release of sodium ions through their chelation of multiple carboxylate groups. Jo et al.^[^ ^53^, ^54^ ^]^ first investigated the potential of EDTA‐4Na (Figure 8c) and DTPA‐5Na (Figure 8d) as sacrificial sodium sources in sodium‐ion batteries. CV curves under dynamic conditions revealed that EDTA‐4Na and DTPA‐5Na undergo oxidative decomposition at 3.79 V (Figure 8e) and 3.61 V (Figure 8f), respectively. The first‐cycle charge curves demonstrated that, with a cutoff voltage of 4.5 V and 4.3 V, these compounds provided charge capacities of up to 420 mAh g^−1^ and 363 mAh g^−1^, respectively. It is worth noting that during the electrochemical oxidation decomposition process, the breakdown of both EDTA‐4Na and DTPA‐5Na produces specific organic groups, including N‐CH_2_‐CH_2_‐N‐2[CH_2_‐COO‐], N‐CH_2_‐CH_2_‐N‐CH_2_‐COO‐ and CH_2_‐OO‐. These groups subsequently bond with nitrogen atoms to form CO and H_2_O. The resulting water molecules then undergo oxidation and decomposition in subsequent electrochemical reactions, contributing to the overall capacity and ultimately leading to a percentage availability exceeding 100%.  When EDTA‐4Na was combined with a P2‐type Na_0.67_[Al_0.05_Mn_0.95_]O_2_ electrode, the charge capacity increased from 83 mAh g^−1^ to 177 mAh g^−1^. Similarly, using DTPA‐5Na as a pre‐sodiation additive in a sodium‐deficient Na_0.44_MnO_2_ electrode increased the capacity from 58 mAh g^−1^ to 128 mAh g^−1^. Furthermore, the C‐N compound of C_3_N formed during oxidative decomposition has been shown to establish a conductive network within the electrode, enhancing electronic conductivity and sodium‐ion diffusion. Notably, the presence of this C‐N compound in DTPA‐5Na composite electrodes resulted in improved initial charge capacity, coulombic efficiency, and capacity retention rate (83% after 500 cycles). These findings highlight the potential of nitrogen‐containing chelating agents as pre‐sodiation additives for enhancing the performance of sodium‐ion batteries.

The precise control over oxidative decomposition potential enabled by molecular engineering strategies for organic sodium carboxylate additives is crucial for optimizing their pre‐sodiation performance. However, the introduction of large functional groups that do not provide active sodium ions can limit the effective compensation capacity of these additives. This challenge has driven the development of novel pre‐sodiation additives featuring multiple sodium carboxylate groups (>2) and optimized decomposition potentials through molecular engineering approaches. By balancing decomposition voltage and sodium compensation capacity, polycarboxylate sodium additives have been shown to be efficient organic pre‐sodiation materials, exhibiting improved overall pre‐sodiation performance.

### 3.2 Polycyclic Arylsodium Compounds for Orangic Chemical Pre‐Sodiation

**Figure 9:**
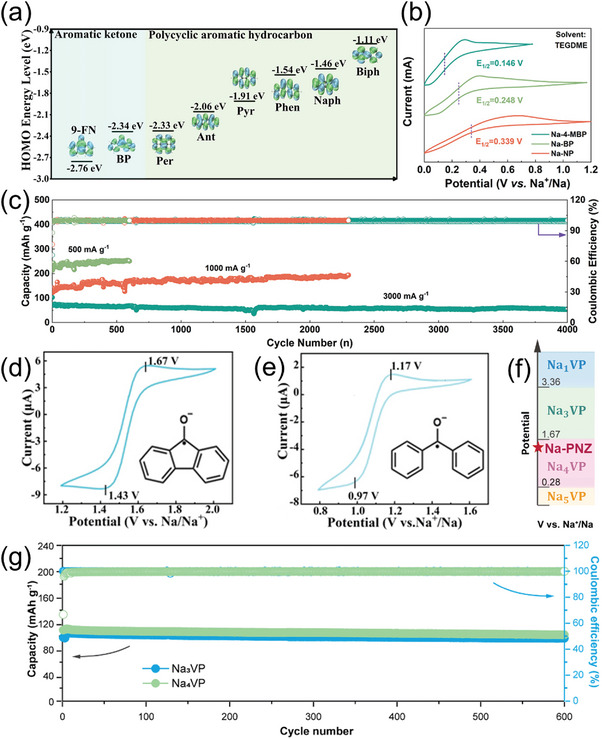
a) HOMO energy levels of anionic radicals of PAHs and aromatic ketones.^[^ ^55^ ^]^ Copyright 2024, Wiley‐VCH. b) CV curves of NP, BP, and 4‐MBP in 0.5 M NaPF_6_ solution. c) Long‐term cycling stability of pNa‐HC at 500, 1000, and 3000 mA g^−1^.^[^ ^27^ ^]^ Copyright 2024, Wiley‐VCH. Cyclic voltammetry curve of d) Na‐9‐FN‐DME and e) Na‐BP‐DME. ^[^ ^55^ ^]^ Copyright 2024, Wiley‐VCH. f) Diagram of the potential matching principle for cathode chemical pre‐sodiation of Na_3_VP. g) Long‐term cycling stability at 1C of Na_3_VP and Na_4_VP electrodes.^[^ ^57^ ^]^ Copyright 2023, Royal Society of Chemistry.

The development of chemical pre‐sodiation technology has witnessed significant advancements in recent years, attributed to its simplicity, uniform reaction mechanisms, and controllable processes. The strong reductive capabilities of polycyclic arylsodium compounds enable rapid electron transfer and sodium ion supplementation to electrode materials, thereby facilitating chemical pre‐sodiation. This approach can be classified into anode and cathode chemical pre‐sodiation, depending on the type of electrode material. Similar to the molecular engineering strategies used for sodium carboxylate additives, polycyclic arylsodium compounds can be functionalized with specific groups to modify the electronic cloud density, thereby adjusting their reductive potential (**Table** **5**). Crucially, this potential tuning must align with the sodium intercalation potential of the electrode material, adhering to the “redox potential matching principle”.^[^ ^31^ ^]^ Changes in the HOMO energy level quantitatively reflect variations in the reductive potential of polycyclic arylsodium compounds.  As demonstrated by Zhang et al.,^[^ ^55^ ^]^ measurement of the HOMO energy levels of commonly used polycyclic aromatic anion radicals reveals a direct correlation between the HOMO energy level and the reductive potential (**Figure** **9**a). A lower HOMO energy level indicates a higher reductive potential for the polycyclic arylsodium compounds, and vice versa.

**Table 5:** Pre‐sodiation properties of various polycyclic arylsodium compounds (Chemical pre‐sodiation).

Materials	Substituent	HOMO energy/eV	Reduction voltage based on CV curve/V	Pre‐sodium Electrode	Cell cycle performance: Capacity retention (%) /Cycle number/ Discharge current c)	Refs.
4‐methylbiphenyl/TEGDME (4‐MBP)	‐CH_3_	−2.252	0.146	Hard carbon	83.72/100/1 (Na_3_V_2_(PO_4_)_3_ ‖ pNa‐HC)	[27]
9‐Fluorenoneb/DME (9‐FN)	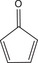	−2.76	1.55	Na_0.44_MnO_2_	72/1000/2 (PS‐NMO ‖ Na)	[55]
Benzophenone/DME (BP)	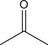	−2.34	1.07	Na_0.44_MnO_2_	56.7/800/2 (PS‐NMO ‖ Na)	[55]
Benzophenone/Toluene (BP)		∖	∖	Na_0.37_Fe[Fe(CN)_6_]_0.86_□_0.14_ · 3.01 H_2_O	70/100/1 (H‐PB ‖ HC)	.^[^ ^56^ ^]^
phenazine‐sodium/DME (PNZ‐Na)	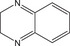	∖	1.56	Na_3_V_2_(PO_4_)_3_	78/100/1 (Na_4_VP ‖ HC)	[57]
			1.6	Na_0.44_MnO_2_	70/195/0.5 (PS‐NMO ‖ HC)	[58]

“∖” represents not mentioned in the references.

The pre‐sodiation of anode materials with low sodium insertion potentials,  such as hard carbon,^[^ ^59^, ^60^, ^61^, ^62^ ^]^ tin,^[^ ^63^ ^]^ titanium,^[^ ^64^ ^]^ antimony,^[^ ^65^ ^]^ phosphorus,^[^ ^66^ ^]^ and reduced graphene oxide (rGO),^[^ ^67^ ^]^ remains a crucial challenge in the development of high‐performance sodium‐ion batteries. The most commonly employed polycyclic arylsodium compounds for this purpose are Na‐biphenyl and Na‐naphthalene, which exhibit relatively strong reducing properties. However, the pre‐sodiation efficiency is significantly influenced by the redox potential of the reducing agent, making it challenging to achieve complete chemical pre‐sodiation (100% ICE) for anodes with extremely low sodium insertion potentials.  To overcome this hurdle, introducing electron‐donating groups into the molecular structure of polycyclic arylsodium compounds is a promising approach. This strategy has been successfully employed by Man et al.,^[^ ^27^ ^]^ who modified the molecular structure of Na‐biphenyl to further lower their reduction potential. Specifically, the introduction of methyl functional groups into the molecular structure of biphenyl and its sodium analogs increased electron density while reducing electron affinity, thereby lowering the reduction potential (Figure 9b). The resulting compounds, including 4‐methylbiphenyl (4‐MBP), exhibited enhanced reducing capabilities, enabling efficient pre‐sodiation of anodes with low sodium insertion potentials. Electrochemical tests demonstrated that hard carbon pre‐sodiated with 4‐MBP (pNa‐HC) achieved a high ICE of 99.1%, along with notable improvements in rate performance and cycling stability. The long‐term performance of pNa‐HC was also excellent, maintaining a capacity of 193.39 mAh g^−1^ after 2300 cycles at a current density of 1000 mA g^−1^ (Figure 9c). Furthermore, when paired with Na_3_V_2_(PO_4_)_3_ cathodes, the full cell exhibited an ICE of 91.25%, further validating the effectiveness of chemical pre‐sodiation in enhancing battery performance. In addition, the positioning of electron‐donating alkyl group substitutions on the benzene ring has been found to play a crucial role in controlling reduction potential. Jang et al.^[^ ^68^ ^]^ explored the effect of methyl substitutions at various positions on the biphenyl (BP) ring, focusing on the redox potential and chemical pre‐lithiation of SiO_x_ anodes. Their study revealed that substituting a single methyl group at the ortho position resulted in a more significant shift in the reduction potential, with an E_1/2_ value of 131 mV for 2‐methyl BP. In contrast, adding two methyl groups at either the meta or para positions (resulting in 3,3′‐dimethyl BP and 4,4′‐dimethyl BP, respectively) led to a lesser reduction potential shift, with E_1/2_ values of 294 mV and 186 mV, respectively. Furthermore, substituting two methyl groups at the ortho position (resulting in 2,2′‐dimethyl BP) lowered the reduction potential below 0 V versus Li/Li^+^, underscoring the significant impact of substituent position on redox behavior.

In contrast to anode pre‐sodiation, where reducing agents with optimal redox potentials are crucial, chemical pre‐sodiation strategies employed to supplement active sodium sources or prepare sodium‐rich cathodes in SIBs require careful consideration of the reduction potential range. Over‐reduction can lead to cathode material decomposition and structural degradation due to over‐sodiation. To mitigate these issues, Zhang et al.^[^ ^55^ ^]^ introduced two carbonyl‐modified aromatic compounds, 9‐fluorenone (9‐FN) and benzophenone (BP), as mild pre‐sodiation reagents for cathodes in SIBs. The incorporation of carbonyl groups (C = O) into PAHs effectively balanced conjugation and inductive effects, resulting in controlled redox potentials.  Specifically, 9‐FN, with a redox potential of 1.55 V (Figure 9d), demonstrated superior performance in pre‐sodiating Na_0.44_MnO_2_ (NMO) and Na_3_V_2_(PO_4_)_3_ (NVP) cathodes. For instance, pre‐sodiated NMO (PS‐NMO) exhibited a discharge capacity of 92.8 mAh g^−1^ with a capacity retention of 72.1% after 1000 cycles at 2C. Additionally, PS‐NVP displayed a significant 145.8% increase in initial charge capacity, reaching 147 mAh g^−1^, and retained 89% of its capacity after 1000 cycles. In contrast, BP, with a lower redox potential of 1.07 V (Figure 9e), showed improved charge capacity but inferior long‐term cycling stability compared to 9‐FN. Zheng et al.^[^ ^56^ ^]^ have recently demonstrated the efficacy of sodium benzophenone as a pre‐sodiation and dehydration treatment for Prussian blue analog (PBA) nanostructures, thereby significantly enhancing the electrochemical performance of  SIBs. This innovative approach addresses two primary challenges associated with PBA‐based electrodes: low sodium content and high crystal water content. By increasing the sodium content within the PBA structure while removing crystal water, the treatment leads to improved electrochemical properties. The treated H‐PB electrodes exhibit an ICE of 98.4% at 0.2 C, with notable improvements in capacity retention (70% after 100 cycles at 1 C) compared to untreated L‐PB electrodes. These enhancements are attributed to the pre‐sodiation and dehydration process facilitated by sodium benzophenone, which ensures enhanced charge/discharge cycling. Furthermore, the H‐PB electrodes demonstrate superior cycling stability and rate performance, underscoring the method's effectiveness in enhancing the overall performance of PBA‐based electrodes for practical SIB applications. These findings underscore the crucial role of carbonyl modification in preventing over‐sodiation and structural degradation, highlighting the promise of 9‐FN and BP as pre‐sodiation reagents for enhancing SIBs cathode performance.

In the previous discussion, we examined the approach of modulating the pre‐sodiation potential via carbonyl modification. Notably, complementary approaches have emerged, featuring alternative pre‐sodiation agents with promising results in cathode chemical pre‐sodiation. Specifically, phenazine derivatives have demonstrated significant potential in enhancing SIBs performance. The application of phenazine sodium (PNZ‐Na) as a pre‐sodiation reagent has been explored by Xu et al.^[^ ^57^ ^]^ The unique electronic structure of PNZ‐Na, featuring nitrogen atoms in an aromatic system, enables it to effectively lower its HOMO energy level and achieve efficient charge dispersion.^[^ ^68^, ^69^, ^70^ ^]^ This property makes PNZ‐Na an ideal candidate for cathode chemical pre‐sodiation, as its low HOMO energy level ensures a well‐matched redox potential with the target material. In their study, Xu et al. employed PNZ‐Na to precisely convert Na_3_V_2_(PO_4_)_3_ (Na_3_VP) into Na_4_V_2_(PO_4_)_3_ (Na_4_VP) in just 90 s, avoiding over‐sodiation through redox potential matching (Figure 9f). The resulting Na_4_VP cathode exhibited a significantly higher energy density of 251.1 Wh kg^−1^ compared to Na_3_VP's 159.4 Wh kg^−1^, along with excellent cycling stability and rate performance. Notably, the Na_4_VP cathode retained 91.8% of its capacity after 600 cycles at 1C (Figure 9g) and maintained high capacity even at higher discharge rates. Furthermore, Kapaev et al.^[^ ^58^ ^]^ present a rapid and scalable chemical pre‐sodiation method using PNZ‐Na to enhance the electrochemical performance of Na_0.44_MnO_2_ cathodes in SIBs. This method significantly boosts the material's capacity and energy density by increasing the sodium content within its structure while minimizing undesirable irreversible reactions. The PS‐NMO shows an 80% increase in specific capacity and a 66% increase in energy density compared to the pristine material. Notably, the pre‐sodiation process with PNZ‐Na minimizes structural degradation and preserves stable cycling performance, offering a promising alternative to traditional electrochemical pre‐sodiation techniques. Additionally, the pre‐sodiation leads to improved ICE and better capacity retention over cycles. These findings demonstrate the effectiveness of PNZ‐Na in enhancing the energy density and cycling stability of sodium‐ion batteries through a rapid and scalable pre‐sodiation process, highlighting its potential as a valuable tool for optimizing cathode performance.

The development of chemical pre‐sodiation strategies using liquid‐phase polycyclic arylsodium compounds has emerged as a promising approach to facilitate the rapid and uniform introduction of sodium ions into electrodes awaiting sodiation. By leveraging molecular engineering principles, it is possible to fine‐tune the reduction potential of these compounds through functional group modification, thereby enhancing their compatibility with target electrodes. This paradigm shift is exemplified by the recent work of Fang et al.,^[^ ^71^ ^]^ who employed a carbonyl‐modified sodium diphenyl ketone (Na‐DK) with a relatively high reduction potential for the chemical pre‐sodiation of HC anodes. By exploiting the reactivity of Na‐DK with oxygen‐containing functional groups and five/seven‐membered ring defects in HC, the authors demonstrated that irreversible sodium consumption can be compensated, leading to the generation of quasi‐metallic sodium within the HC structure. In the context of anode chemical pre‐lithiation, comparable studies have been conducted. For instance, Li et al.^[^ ^72^ ^]^ explored electron‐withdrawing cyano‐modified 1‐cyano‐naphthalene as a bifunctional chemical pre‐lithiation agent for SiO anodes. This approach enabled the achievement of controllable pre‐lithiation of the SiO electrode, with ICE reaching 100%. Moreover, the introduction of the negatively charged cyano group resulted in a milder pre‐lithiation reaction, which had dual benefits. First, it prevented the active material from detaching from the copper current collector, thereby maintaining structural integrity. Second, it facilitated the formation of a functionally integrated interface film on the SiO‐based electrode, optimizing interfacial interactions. These findings challenge the current assumption that reduction potential is the sole determining factor for chemical pre‐sodiation, underscoring the need for a deeper understanding of the underlying mechanisms through interdisciplinary collaboration and advanced in situ characterization techniques. Furthermore, the molecular engineering strategy based on electron‐donating functional group modifications can also be applied to enhance the reduction ability of organic sodium reagents. Notably, Shen et al.^[^ ^73^ ^]^ provided a reference case in a similar chemical pre‐lithiation field. DFT calculations revealed that Li‐BP in the electron‐donating methyl‐modified 2‐methyltetrahydrofuran (2‐Me‐THF) solvent system exhibits the highest HOMO energy level compared to tetrahydrofuran (THF) and dimethoxyethane (DME) solvent systems. The Li‐BP/2‐Me‐THF system, with an oxidation‐reduction potential as low as 0.08 V, provides a better lithium compensation effect when conducting chemical pre‐lithiation on graphite anode materials with low lithium intercalation potential. Moreover, the persistent issues of high cost and residual organic reagents in chemical pre‐sodiation underscore the necessity for developing efficient post‐treatment systems and identifying cost‐effective precursor molecules.

## 4 Conclusion and Outlook

Sodium‐Ion Batteries have emerged as promising alternatives to lithium‐ion batteries due to the abundance and cost‐effectiveness of sodium resources. However, SIBs encounter significant challenges related to substantial active sodium ion losses during electrochemical cycling, primarily caused by solid electrolyte interphase formation, anode expansion, and side reactions. These issues severely impact the electrochemical performance of SIBs, particularly the initial Coulombic efficiency and cycling stability. To address these challenges, pre‐sodiation technology has been introduced to artificially supplement active sodium sources within the SIBs system. Organic compounds have emerged as a promising class of materials in the development of pre‐sodiation technologies due to their inherent design diversity, allowing for the creation of various structures through different bonding methods and the introduction of diverse functional groups. These functional groups can modulate reactivity, polarity, and electrochemical characteristics, rendering organic compounds an attractive option for pre‐sodiation. The versatility of organic compounds in pre‐sodiation technology can be categorized into two operational principles: organic additives and chemical pre‐sodiation. Crucial to the efficient operation of these technologies is precise control over oxidative decomposition and reduction potentials.

Molecular engineering strategies are essential for regulating the potential of organic molecules, with functional group modification being an effective means of altering their properties. In recent years, researchers have employed various modification techniques to optimize the performance of organic pre‐sodiation molecules. These efforts have led to the development of versatile molecular modification strategies aimed at optimizing molecular properties for efficient pre‐sodiation. However, a comprehensive review that systematically summarizes the molecular engineering strategies used in organic pre‐sodiation is still lacking. This review aims to provide a comprehensive examination of molecular engineering strategies employed in organic pre‐sodiation technology, emphasizing both fundamental principles and practical implementations. Furthermore, recent advances in the application of organic additives and chemical pre‐sodiation were summarized, integrating the most up‐to‐date research findings.

**Figure 10:**
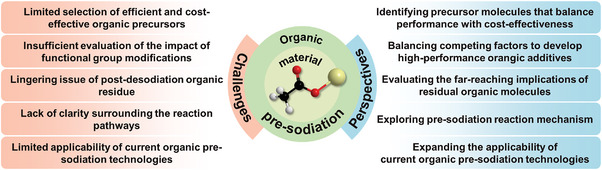
Future perspectives of organic pre‐sodiation technology.

Despite notable advancements in organic pre‐sodiation techniques, several key challenges still need to be overcome for further optimization and development. Specifically, a limited selection of efficient and cost‐effective organic precursors, insufficient evaluation of the impact of functional group modifications, lingering issue of post‐desodiation organic residue, lack of clarity surrounding the reaction pathways and limited applicability of current organic pre‐sodiation technologies require continued investigation to fully unlock the potential benefits of application potential of organic materials for pre‐sodiation. In light of these challenges, we propose several prospective strategies aimed at advancing organic pre‐sodiation technology. Moreover, critical knowledge gaps that require further investigation are identified (**Figure** **10**), with the ultimate goal of guiding future innovations and facilitating the continued development of organic pre‐sodiation technologies.

1. Identifying precursor molecules that balance performance with cost‐effectiveness. Despite the superior performance and functional advantages of certain organic molecules, such as arylsodium reagents and squaric acid‐based additives, their high production costs have hindered widespread adoption in large‐scale applications. The economic burden of these materials often outweighs their potential benefits, highlighting the need for low‐cost alternatives. To address this challenge, future research should focus on identifying and evaluating precursor molecules that balance performance with cost‐effectiveness. The integration of machine learning and high‐throughput computing has revolutionized the screening of pre‐sodiation organic molecules, enabling efficient analysis of large molecular datasets and rapid identification of high‐performance, cost‐effective materials. Moreover, innovations in biotechnology, process optimization, and sustainable manufacturing practices offer opportunities for cost reduction. By leveraging these advancements, researchers can identify materials that balance both performance and economic feasibility, ultimately facilitating the widespread adoption and commercialization of organic molecules in pre‐sodiation applications.
2. Balancing competing factors to develop high‐performance organic additives. The optimization of organic pre‐sodiation molecules is inherently complex, as improvements in one parameter may come at the expense of others. The combined trade‐offs principle highlights the need for balancing competing factors to develop high‐performance molecular additives. In this context, the utilization of long‐chain alkyl groups, aromatic rings, and other bulky substituents has shown promise in modifying organic pre‐sodiation molecules. However, the steric hindrance effects associated with these large substituents remain underappreciated in the context of organic pre‐sodiation. The presence of these bulky groups can impede orbital overlap, reduce π‐conjugation and electronic delocalization, thereby lowering the HOMO energy level. Furthermore, steric hindrance can introduce conformational stress or changes, influencing intramolecular electron distribution and accessibility. To optimize the performance of organic pre‐sodiation molecules, it is essential to comprehensively consider both inductive effects and steric hindrance when designing bulky substituent modifications. By acknowledging the trade‐offs between functional group modifications, researchers can strategically navigate these complexities to further optimize organic pre‐sodiation molecules, ultimately enhancing battery performance.
3. Evaluating the far‐reaching implications of residual organic molecules. The organic pre‐sodiation technique has been demonstrated to effectively mitigate sodium ion loss during the initial cycle, but its long‐term implications on battery performance, safety, and cycle life must be carefully considered. Specifically, the post‐desodiation behavior of residual organic molecules poses a crucial question: what impact do these residues have on battery systems? Evaluating the far‐reaching implications of residual organic molecules on battery operation and lifespan is crucial to mitigating these risks and ensuring reliable energy storage, encompassing an examination of decomposition mechanisms, product characterization, and interactions with other components. Furthermore, advanced characterization techniques are necessary to accurately monitor and quantify organic residues in the battery, enabling a strategic approach that selects organic molecules whose residues contribute positively to the system's performance.^[^ ^74^ ^]^ By optimizing the pre‐sodiation process to minimize adverse effects and improve overall performance and reliability, researchers can unlock the full potential of sodium‐ion batteries.
4. Exploring pre‐sodiation reaction mechanism. Optimizing desodiation reaction pathways is essential for enhancing the performance of sodium‐ion batteries, particularly with respect to cycle stability and energy efficiency. However, pre‐sodiation reactions often suffer from inefficiencies and low selectivity, highlighting the need for comprehensive mechanistic investigations to underpin optimization efforts. A deep understanding of desodiation mechanisms is crucial for refining pre‐sodiation reaction pathways, which can be achieved through a combination of kinetic analysis, isotopic labeling experiments, and computational chemistry simulations. One such technique is Isotope Transient Kinetic Analysis (ITKA), which offers a unique approach by merging isotope tracing with transient experimental methods. By labeling reactants or intermediates with isotopes, ITKA enables real‐time tracking of dynamic changes throughout the reaction, providing valuable insights into the spatiotemporal evolution of reactants, products, and intermediates. This methodology provides both quantitative and qualitative information on potential reaction mechanisms. In addition to ITKA, in situ X‐ray absorption spectroscopy, in situ infrared spectroscopy, and in situ Raman spectroscopy offers multidimensional data on the reaction mechanism, elucidating structural transformations, electronic state changes, and molecular interactions. These techniques provide critical theoretical and experimental support for understanding the intricacies of pre‐sodiation reactions. Furthermore, Ultrafast Transmission Electron Microscopy offers a powerful tool by combining the high spatial resolution of electron microscopy with the temporal resolution of ultrafast laser pulses. This technique enables real‐time observation and analysis of nanoscale structural alterations, species transformations, and dynamic processes during the reaction. The seamless integration of these advanced characterization techniques with DFT calculations allows for a more profound understanding of the intricate reaction pathways involved in pre‐sodiation. By investigating various pre‐sodiation additives and polycyclic arylsodium compounds, researchers can identify and characterize the rate‐determining steps that govern the reaction mechanism, ultimately enabling the development of more efficient and selective organic pre‐sodiation reactions.
5. Expanding the applicability of current organic pre‐sodiation technologies. The limited applicability of current organic pre‐sodiation technologies presents a significant challenge, as it restricts their widespread adoption and hampers efforts to expand the scope of these innovative approaches to enhance sodium‐ion battery performance. To further solidify its position as a cutting‐edge technology, organic pre‐sodiation must be adapted to address diverse application scenarios and foster systemic interoperability with other disciplines. One promising avenue for enhancing the value of recycled materials is exploring the integration of organic pre‐sodiation with waste battery regeneration processes.^[^ ^31^, ^75^, ^76^ ^]^ This synergy can lead to improved performance and sustainability through the upcycling of previously discarded materials. Furthermore, combining organic pre‐sodiation with the preparation of ion‐intercalated exfoliated nanosheets (e.g., single‐layer graphene^[^ ^77^ ^]^ and MoS_2_ nanosheets^[^ ^78^ ^]^) could unlock new opportunities for interlayer ion exchange and exfoliation efficiency, yielding nanomaterials with superior performance. Moreover, research into utilizing organic pre‐sodiation to enhance the stability and safety of electrode materials can contribute significantly to overall battery safety. By embracing a multidisciplinary approach that brings together experts from diverse fields, we can not only expand the scope of organic pre‐sodiation but also pioneer innovative breakthroughs and advancements in materials science and energy storage and conversion.

In conclusion, the quest for high‐energy‐density sodium‐ion batteries presents a compelling opportunity for innovations in organic pre‐sodiation technologies. While significant progress has been made, future research should prioritize addressing existing technical hurdles and optimizing pre‐sodiation systems to enhance efficiency and cost‐effectiveness. This review provides a comprehensive overview of recent advancements in organic materials utilized for pre‐sodiation, highlighting the most promising developments and offering critical insights into prospective research trajectories. As the field continues to evolve, organic pre‐sodiation technologies are poised to play a pivotal role in achieving high energy density alongside exceptional cycling performance in sodium‐ion batteries. To facilitate commercialization and widespread adoption of these advanced energy storage solutions, future initiatives must tackle essential technical obstacles and refine foundational systems. This review serves as both a theoretical framework and a practical resource for further advancements in organic pre‐sodiation technologies, with the ultimate objective of enhancing the performance and sustainability of sodium‐ion batteries.

## Conflict of Interest

The authors declare no conflict of interest.
